# Sero-prevalence of HTLV 1/2 and HCV in paired mother and children from Malawi as well as systematic review and meta-analysis of HTLV-1/2 and HCV of data available on women and children living in Africa

**DOI:** 10.1186/1742-4690-11-S1-P58

**Published:** 2014-01-07

**Authors:** James M Fox, Robert Newton, Marija Bedaj, John Martin Bland, Nora Mutalima, Fabiola Martin

**Affiliations:** 1 Centre for Immunology and Infection, Department of Biology & Hull York Medical School, University of York, York, North Yorkshire, UK; 2 Department of Health Sciences, University of York, York, North Yorkshire, UK

## 

HTLV prevalence is poorly mapped in several areas, in particular in Africa. Global prevalence is commonly cited as being 10-20 million but more recently this has been contested as an overestimation. Additional epidemiological studies would further our understanding of the HTLV burden. Similarly, HCV is endemic in certain locations (Egypt) whilst data for other areas of Africa are virtually absent. We investigated HTLV/HCV prevalence in serum samples from 418 paired mother and child samples of healthy mothers of children attending Malawian hospitals. Latest generation ELISAs were used; consistently ELISA reactive mothers’ sera were further tested by immunoblot and only deemed sero-positive if they were reactive in this assay. Serum from the children of ELISA positive/borderline mothers were subsequently screened. Sero-prevalence in the healthy women was as follows, HTLV-1: 0.72% (n=3), HTLV-2: 1.9% (n=8), HTLV indeterminate: 3.8% (n=16) and HCV: 0.48% (n=2). DNA extracted from whole blood could only resolve one of the high percentage of intermediate positive results, most likely because of a low proviral load. One child in each category: HTLV-1, HTLV-2, dual-infected and HTLV indeterminate was identified indicating low (11.54%) mother to child transmission (MTCT) whereas one of the two HCV positive mothers had a HCV positive child, suggestive of high HCV MTCT transmission rate. We conducted a systematic review to compare this original data with previous reports: Malawi had similar, low, levels of HTLV-1 and HCV but surprisingly high HTLV-2. Prevalence maps for HTLV and HCV for this demographic in the African continent and the meta-analysis will be presented.

